# Serum Uromodulin as early marker for ischemic acute kidney injury and nephron loss: association with kidney tissue distribution pattern

**DOI:** 10.1186/s12967-025-06125-x

**Published:** 2025-03-14

**Authors:** Eva Vonbrunn, Nadja Ebert, Nada Cordasic, Kerstin Amann, Anke Büttner, Maike Büttner-Herold, Jürgen E. Scherberich, Christoph Daniel

**Affiliations:** 1 Department of Nephropathology, Institute of Pathology, Friedrich-Alexander-Universität Erlangen-Nürnberg, Universitätsklinikum Erlangen, Krankenhausstr. 8-10, 91054 Erlangen, Germany; 2 School of Psychology, College of Life and Environmental Sciences, University of Birmingham, Birmingham, UK; 3 Klinikum Munich-Harlaching, Ludwig-Maximilians University, Munich, Germany

**Keywords:** Uromodulin, Tamm-Horsfall protein, Ischemia/reperfusion, Acute kidney injury, Nephron loss

## Abstract

**Background:**

Uromodulin (UMOD) is expressed in kidneys and is mainly excreted in the urine, although a smaller amount is also released into the serum. Here, we investigated UMOD in acute kidney injury (AKI), with particular focus on the utility of serum UMOD as marker for nephron loss.

**Methods:**

Blood and kidney samples were collected 6 h, 24 h, 3 days and 8 weeks after ischemia/reperfusion (I/R) in a rat model. To investigate the impact of nephron number on UMOD levels, sera and tissue from healthy, uninephrectomized (Unx) and 5/6-nephrectomized (Snx) rats were analyzed. Histological changes, kidney function and cell damage were evaluated and serum UMOD, *Umod* mRNA expression and distribution of UMOD protein in the kidney were examined.

**Results:**

In AKI, kidney function was markedly impaired 24 h after I/R, while kidney injury and serum UMOD was increased transiently. Simultaneously, the amount of UMOD-positive kidney cells rapidly decreased 24 h after I/R compared to healthy kidneys, and mRNA expression of *Umod* was lowest on days 1–3 after I/R. Serum UMOD correlated with nephron number showing the highest levels in healthy rats, which were reduced after Unx and further reduced after Snx.

**Conclusion:**

In an AKI model with severe tubular damage, a transient increase in UMOD serum levels in parallel with loss of UMOD-positive cells suggests temporary release of UMOD from destroyed tubular cells into the blood. Serum UMOD appears to be not only a marker of chronic renal failure but also of acute loss of functional and cellular integrity of kidney epithelia in AKI.

## Background

Uromodulin (UMOD) is a 90 kDa glycoprotein exclusively produced in epithelial cells of the thick ascending limb (TAL) of the loop of Henle [1], nearly completely released from the apical membrane and excreted in the urine (30–60 mg/day) [2, 3]. UMOD protects against bacterial urinary tract infection [4, 5] and plays a role in chronic pyelonephritis and pathogenesis of urolithiasis [6–8]. UMOD reveals various renoprotective characteristics and immunological effects, being either pro- or anti-inflammatory [9].

To a lesser extent, the protein is released basolaterally into the renal interstitium, where its function remains to be elucidated [10, 11]. UMOD serum concentrations relate to eGFR and chronic kidney disease (CKD) [12, 13], an association which is much stronger for serum than for urinary UMOD [14]. In cases with reduced nephron number, such as in CKD, UMOD level decreases in parallel [15–17]. After transplantation, urinary and serum UMOD levels of kidney donors decreased, suggesting that the total biosynthesis of UMOD depends on the number of nephrons [18, 19]. The influence of nephron loss and its accompanying histopathological changes on serum UMOD levels have not been investigated yet.

Little is known about UMOD in acute kidney injury (AKI). Serum UMOD increases in experimental AKI and plays a protective role in ischemic renal injury [20, 21].

The fact that UMOD is a kidney specific protein offers the opportunity to discover new pathophysiological mechanisms regarding the renal microenvironment. Therefore, we aimed to elucidate the link between UMOD serum concentrations, immunohistological distribution and gene expression in the kidneys of rat models with either severe AKI or chronic nephron loss. Additionally, we studied human renal biopsies taken few days after kidney transplantation.

## Material and methods

### Animal models

The protocol for the experimental animal studies was approved by the German regional committee for animal care and use, which is equivalent to the US IACUC, and by governmental authorities (Regierung von Unterfranken, approval numbers 55.2.2-2532-2-1127 for ischemia/reperfusion and 54-2532.1-14/12 for 5/6-nephrectomy). The studies were conducted in strict accordance with the German Animal Welfare Act and using the ARRIVE reporting guidelines [22]. Male WT Dark Agouti rats were bred and housed under specific pathogen-free conditions in the "Preclinical Experimental Animal Center" of the Medical Faculty of the FAU. Surgical procedures and kidney harvesting at the end of the experiments were performed under isoflurane anesthesia and analgesia with 0.05 mg/kg body weight Buprenovet (Bayer AG, Leverkusen, Germany). For the animal experiments, the required group sizes were calculated based on preliminary serum creatinine data and using GPower 3.1 software [23]. Prior to animal testing, termination criteria, such as weight loss greater than 20% of body weight, were established to prevent severe distress to the animals. No animals were withdrawn from the study. To minimize the possible influence of confounding factors, animals from the same cage were assigned to different test groups and the experiments were completed in several rounds.

### Ischemia/reperfusion model (I/R)

**Fig. 1:**
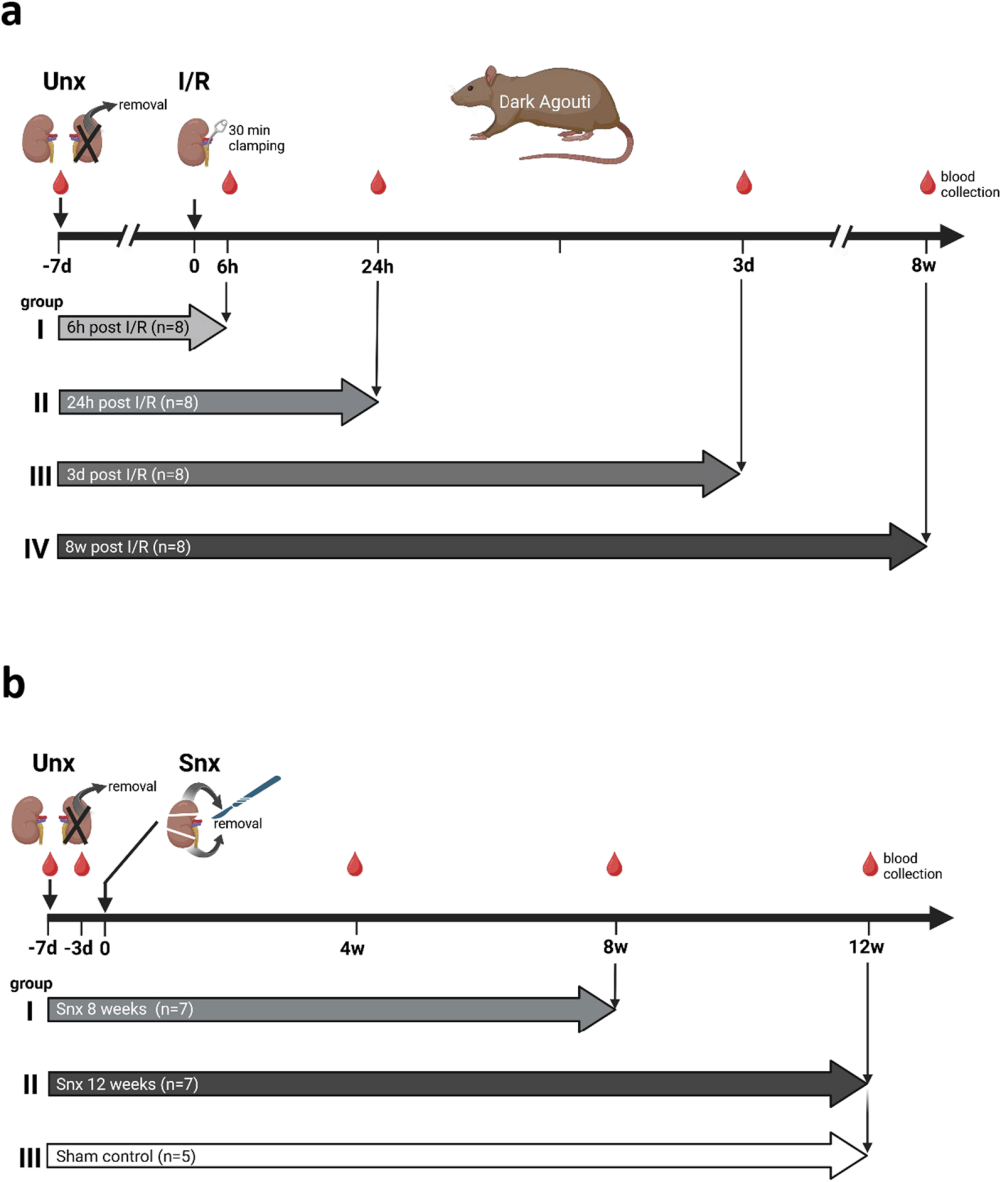
Experimental design of the animal experiments. An overview of the experimental design, procedures and blood sample collection is shown for the ischemia/reperfusion (I/R) (**a**) and 5/6 nephrectomy (Snx) (**b**) models. Kidney tissue was collected at the endpoints for each group. Created in BioRender, Daniel, C. (2024) https://BioRender.com/s47a453 and https://BioRender.com/q59n446)

One week before induction of I/R, the right kidney of Dark Agouti (DA) rats (150–200 g) was removed and served as a healthy control. Ischemia was induced by clamping the renal artery of the left kidney for 30 min while the animals were maintained at 37 °C using a heated operating table equipped with a rectal probe. Rats were sacrificed and kidneys harvested 6 h, 24 h, 3 d and 8 weeks after reperfusion (n = 8 each) for mRNA analysis and (immuno-)histology. Serum samples were collected before removal of the right kidney and at the end of the experiment for UMOD (ELISA) and creatinine analysis. An overview of the experimental design with the procedures that were carried out and the samples that were collected is shown in Fig. 1a.

### 5/6-Nephrectomy model

In order to minimize stress to the animals, the 5/6 nephrectomy was performed in a two-step protocol. First, the right kidney of DA rats (180–280 g) was removed after ligation of the right renal artery, renal vein and ureter. During this procedure, the renal capsule was carefully displaced to preserve the adrenal gland. Second, one week after uni-nephrectomy (Unx), a standardized surgical resection of the renal cortex was performed, with the resected amount equal to 2/3 of the weight of the previously removed right kidney (Snx). The experiment was terminated at 8 (n = 7) and 12 weeks (n = 7). Sham-operated rats were used as controls (n = 5). Kidneys were harvested for (immuno-)histological evaluation. Additionally, blood was collected from the tail vein before (n = 7) and 4 d after Unx (n = 8) and 4 weeks after Snx (n = 9). The first time point before operation served as a normal reference, 4 days after Unx were chosen to study the effects of a 50% reduction in kidney mass. sUMOD has a half-life of approximately 16 h in the rabbit [24], thus four days after halving the kidney mass less than 2% of the UMOD produced by the removed kidney should remain. The second time point of 4 weeks after Snx was chosen to observe the effects of a 5/6 reduction in kidney mass before massive changes in kidney morphology. Such effects as extensive renal fibrosis, and a pronounced inflammatory response are only present at later time points, e.g. at 8 and 12 weeks. An overview of the experimental design, procedures and sample collection is shown in Fig. 1b.

### Human renal transplant biopsies

Kidney biopsies were taken after renal allotransplantation at the Department of Nephrology and archived at the Department of Nephropathology (both University Hospital, Erlangen, Germany) after use for routine diagnosis. The use of remnant kidney biopsy material has been approved by the Ethics Committee of the Friedrich-Alexander University of Erlangen-Nuremberg, waiving the need for retrospective consent for the use of archived excess material (Ref. No. 22-150-D). The human biopsies were obtained within the first 10–24 days post transplantation from patients with impaired renal function necessitating dialysis within the first 10 days after transplantation (delayed graft function, DGF) (n = 6). One year protocol biopsies with normal function of kidney grafts and no evidence of rejection (n = 7) served as controls. Detailed information about the cohort has been previously provided by Vonbrunn et al. [25].

### Assessment of histologic renal changes

Kidney sections were stained with periodic acid-Schiff (PAS) stain and analyzed for acute tubular injury (ATI), tubular atrophy, interstitial fibrosis and glomerulosclerosis using a semi-quantitative score. At least 10 high-power fields at 200 × magnification were analyzed applying scores of 0 to 4, corresponding to none (0), up to 25% (1), 25–50% (2), 51–75% (3) and more than 75% histologic changes (4). Finally, mean scores were calculated for each kidney section and parameter. The evaluation was conducted blinded.

### Immunohistochemistry and immunofluorescence staining

Immunohistochemistry for UMOD and immunofluorescence staining of UMOD, KIM-1, ED1 and CD3 was performed as described previously [25, 26]. FFPE kidney sections were deparaffinized, rehydrated, blocked for endogenous peroxidase activity followed by antigen retrieval with target retrieval solution pH 6 (DAKO Deutschland, Hamburg, Germany) for 2.5 min in a pressure cooker. For IHC staining, an avidin–biotin blocking kit (Vector Laboratories, Burlingame, CA, USA) was applied and sections were layered with the primary antibody against UMOD (mouse monoclonal anti-rat; Santa Cruz Biotechnology; 1:1000 or mouse monoclonal anti-human, clone T112A provided by Dr. J. Scherberich; 1:2000) overnight at 4°C. After washing with wash buffer (50 mM Tris pH 7.4 supplemented with 0.1% (v/v) Tween 20), biotinylated secondary antibody (biotinylated anti-mouse IgG; Vector Laboratories, 1:500) was added and incubated for 30 min at RT. ABC-Kit and Vectastain DAB Immpact Kit (both Vector Laboratories) were used to visualize the bound secondary antibody. For negative controls, immunostaining was performed by omission of the primary antibody. The UMOD distribution in the kidney was evaluated in 20 high resolution fields per kidney section, of which 5 were observed in the medulla, 5 in the inner stripe, 5 in the outer stripe and 5 in the cortex at 200 × magnification. The number of UMOD-expressing tubules and intratubular casts were counted and converted to an area of 1 mm^2^.

For immunofluorescence double-staining of UMOD (mouse monoclonal anti-rat; Santa Cruz Biotechnology; 1:2000), KIM-1 (goat polyclonal anti-rat; R&D; 1:1000), ED1 (mouse monoclonal anti-rat; Bio-Rad Laboratories; 1:50) and CD3 (rabbit monoclonal anti-rat; Zytomed Systems GmbH; 1:50), primary antibodies were applied directly after antigen retrieval, and were incubated over-night at RT. After washing with wash buffer, secondary antibodies (goat anti-mouse IgG1 Alexa 488; Dianova, goat anti-rat IgG Alexa Fluor; Thermo Fisher, goat anti-rabbit IgG Alexa 750; Thermo Fisher) were diluted 1:500 and incubated for 30 min at RT in the dark. Sections were counterstained and coverslipped with Vectashield Vibrance (Vector Laboratories). Slides were imaged on the Axio Scan.Z1 slide scanner (Zeiss, Oberkochen, Germany) and analyzed using QuPath version 0.2.3 software [27]. The wand tool was used to detect and localize the entire tissue area, and the percentage of positively stained cells was calculated using the object classifier. Hereby, UMOD-expressing cells could be differentiated from UMOD-positive casts. The profile tool of the ZEN software (Blue edition, version 3.3) was used to analyze the distribution and intensity of the fluorescence signal.

### UMOD ELISA

For quantification of UMOD in rat plasma an in vitro Uromodulin ELISA (Rat Uromodulin SimpleStep ELISA Kit; Abcam, Cambridge, UK) was used according to the manufacturer's instructions and measured using the Synergy 2 plate reader and Gen5 software (both from BioTek Germany, Bad Friedrichshall, Germany) at a wavelength of 450 nm.

### Immunoprecipitation and Western blot analysis of rat UMOD

For analysis of UMOD in rat serum and urine, immunoprecipitation was first performed with an agarose conjugated anti-UMOD antibody (mouse monoclonal anti-rat; Santa Cruz Biotechnology; 10 µg/ml). The pellet containing the precipitated UMOD was then boiled for 5 min in sample solution and applied to a 7.5% SDS-PAGE along with a protein marker (ProSieve QuadColor Protein Marker, 4.6–300 kDa; Biozym Scientific, Hessisch Oldendorf, Germany). Proteins were then transferred to a nitrocellulose membrane by Western blotting. A mouse monoclonal anti-rat antibody (Santa Cruz Biotechnology; 1:100) was used to detect UMOD, followed by visualization with a goat anti-mouse secondary antibody (Thermo Fisher Scientific, Waltham, USA; 1:10,000) and Immobilon Western HRP substrate (Merck, Darmstadt, Germany).

### Multiplex mRNA expression analyses

*Umod* mRNA expression was analyzed using NanoString nCounter technology and a custom code set for rat renal tissue and the Human Organ Transplant Panel for human biopsies (both NanoString Technologies, Seattle, WA) as described earlier [25]. After homogenization of 20 mg of fresh-frozen rat kidney tissue using Precelly tubes, a homogenizer (VWR, Radnor, PA, USA) and QIAshredder spin columns (Qiagen, Venlo, The Netherlands), total RNA was isolated using the RNeasy Mini Kit (Qiagen) according to the supplier's protocol. RNA isolation from human biopsies was performed using 15 µm FFPE tissue sections and the RNeasy FFPE Kit (Qiagen). RNA concentration and purity were measured using NanoDrop spectrophotometry (Thermo Fisher). RNA was diluted with H_2_O to a volume of 5 µl containing 100–200 ng RNA and afterwards hybridized with NanoString probes for 20 h. A NanoString custom code set including Umod was used for gene expression analysis of rat kidneys and the Human Organ Transplant Panel for human biopsies (both NanoString Technologies, Seattle, WA). Preparation and multiplex gene analysis were performed according to the manufacturer's recommendations with the NanoString nCounter FLEX analysis system (NanoString Technologies). Quality control and data normalization were conducted using nSolver Analysis Software Version 4.0 and the internal negative control probes, synthetic positive controls and housekeeping genes of the particular panels.

### Statistical analysis

Values were tested for outliers using the ROUT outlier test with Q = 1%. Normal distribution was tested using the Kolmogorov–Smirnov test. Comparisons of more than 2 groups were analyzed using a one-way analysis of variance (ANOVA) followed by Tukey`s multiple comparison test or, if not normally distributed, a Kruskal–Wallis test followed by Dunn`s multiple comparison test. Significant differences between 2 groups were evaluated using the Mann–Whitney U rank test or the Welch’s t-test. GraphPad Prism 8.0 was used for calculations; *P* value < 0.05 was considered statistically significant (* *P* < 0.05; ** P < 0.01; *** P < 0.001; **** P < 0.0001).

## Results

### Serum UMOD transiently increased in acute kidney injury

**Fig. 2:**
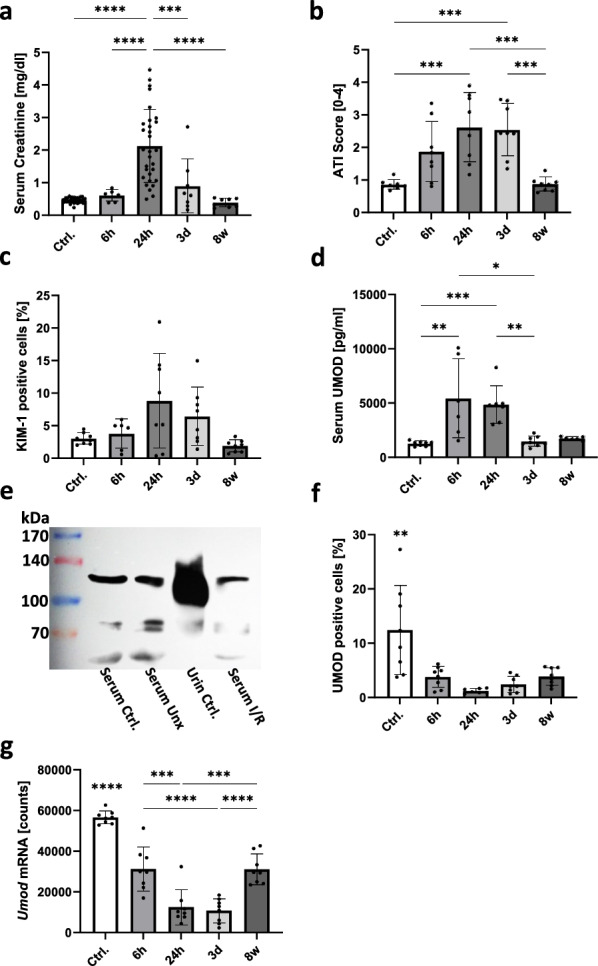
Kidney function, injury and UMOD/*Umod expression* in serum and renal tissue in an I/R rat model. Kidney function was assessed by serum creatinine [mg/dl] (**a**); Acute tubular injury (ATI) was analyzed by scoring of histological changes (**b**) and kidney injury was measured as percentage of KIM-1 positive cells in IF staining (**c**); serum UMOD was measured using ELISA (**d**); comparison of serum from rats 24 h post I/R to serum and urine UMOD of healthy rats by Western Blot (**e**); Percentage of UMOD positive cells in IF stained rat renal tissue (**f**) and number of *Umod* mRNA molecules assessed by gene expression analysis (**g**). All analysis were conducted at different time points: before I/R and 6 h, 24 h, 3 d and 8 w post I/R; (* *P* < 0.05; ** *P* < 0.01; *** *P* < 0.001; **** *P* < 0.0001)

Serum creatinine was highly significantly elevated 24 h after I/R (fourfold compared to controls) and recovered rapidly during the following days (Fig. 2a). ATI as well as KIM-1 as a marker of kidney injury increased starting 6 h post-reperfusion, but also peaked at 24 h, followed by a longer recovery period of a few days until complete regeneration at 8 weeks (Fig. 2b, c). Serum UMOD already markedly increased 6 h after I/R (fivefold compared to controls) and remained at a similarly high level for 24 h before completely normalizing on day 3 after I/R (Fig. 2d), correlating with serum creatinine levels but not with the degree of ATI (Table 1).

**Table 1:**
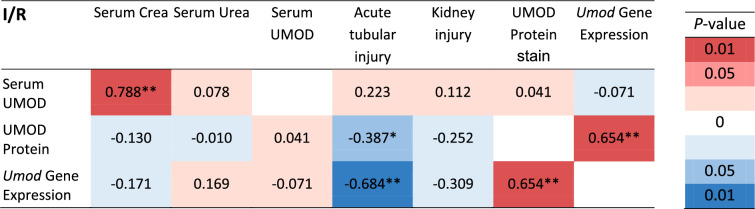
Overview of the correlation between UMOD/*Umod* expression and kidney injury in IRI

The correlation coefficients of all correlation analysis between results for serum UMOD, UMOD protein (UMOD-positive cells detected by immunofluorescence staining) and *Umod* gene expression and evaluation of kidney injury were summarized and displayed in a heat map; n = 28 respectively n = 40 (* *P* = 0.05; ** *P* = 0.001). Positive correlations are colored red, negative correlations blue, and highly significant correlations are colored deeper

To test whether urinary reflux of UMOD may cause the transient increase in serum uromodulin levels, UMOD was analyzed by Western blot analysis in serum samples from healthy and Unx rats and rats 24 h after I/R as well as in urine from healthy rats. Full-length UMOD with an estimated molecular weight of 120 kDa was detected in all serum samples. Even in the serum sample collected 24 h after I/R no cleaved UMOD could be detected, which lacks the external hydrophobic patch (EHP) sequence and is approx. 20 kDa smaller than full-length UMOD. The latter represented the dominant urinary UMOD (Fig. 2e). Bands at 75 kDa presumably represent UMOD degradation products, which were detected in similar amounts in all samples. An additional band at 60 kDa was present only in serum samples (Fig. 2e).

### UMOD-positive renal cells and renal *Umod* gene expression are markedly reduced in an I/R model of AKI

In kidney tissue after I/R, the percentage of UMOD-positive cells decreased continuously over time in the control group from 12.4 ± 7.7% to 1.8 ± 1.8% in samples taken 24 h after reperfusion and hardly regenerated within the following 3 days (Fig. 1f). However, 8 weeks later the number of UMOD-positive cells increased again slightly to 3.8 ± 1.5% (Fig. 2f).

*Umod* gene expression was markedly reduced 24 h after reperfusion by more than 75% compared to healthy controls (Fig. 2g). Eight weeks later the gene expression substantially recovered and reached 2/3 of the initial expression quantity (Fig. 2g). Although the number of UMOD-positive cells in AKI animals was only one-third of the controls at the same time point (Fig. 2f, 8w), it appeared that residual surviving epithelia of the TAL segment expressed more *Umod* at single cell level (Fig. 2g, 8w). Interestingly, the number of UMOD-positive cells and *Umod* expression did not correlate with serum creatinine but showed significant negative correlation with the degree of kidney cell injury (Table 1).

### Distribution pattern of kidney tissue UMOD during acute kidney injury

**Fig. 3:**
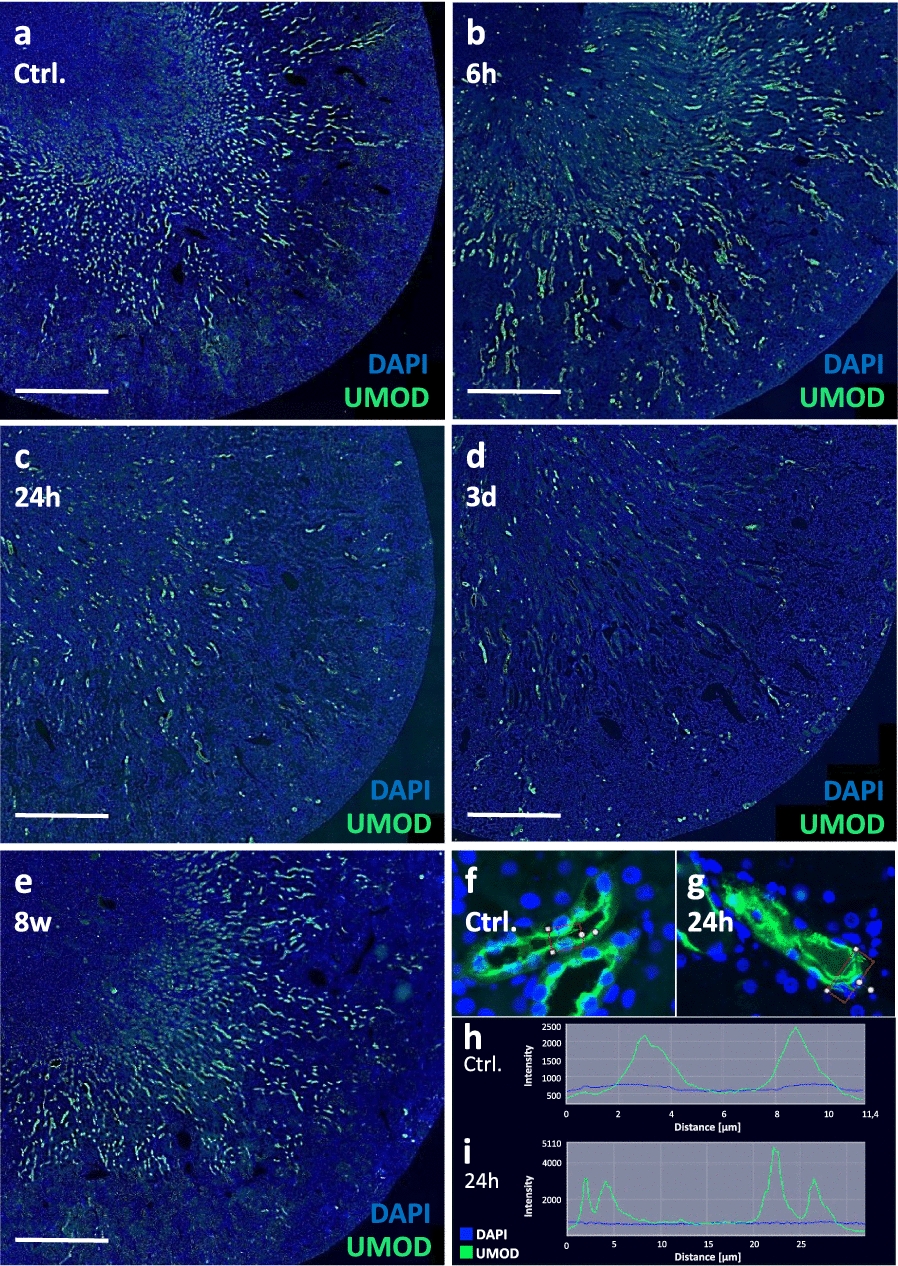
Immunofluorescence staining of UMOD in I/R kidneys. Examples of the distribution of UMOD protein in kidneys from healthy rats (Ctrl.; **a**) and rats with I/R at different time points (6 h, 24 h, 3 d and 8 weeks) post reperfusion (**b–e**); Measurement of fluorescence signal distance and intensity in tubules of Ctrl. group (**f**, **h**) and 24 h after I/R (**g**, **i**); Staining: blue = nuclei; green = UMOD; Scale bar = 1 mm

Not only the amount but also the distribution of UMOD in the different compartments of the kidney changed over time after I/R. While in healthy kidneys UMOD was mainly detected radially in the inner and outer stripe (Fig. 3a), after 6 h the first UMOD-positive tubular protein casts appeared in the medulla and cortex (Fig. 3b). Even so, the original distribution of the antigen in cells of the inner and outer stripe was still visible. This pattern vanished after 24 h and tubular UMOD positivity and casts were almost evenly distributed between medulla and inner and outer stripe, with slightly fewer UMOD-positive cells in the cortex (Fig. 3c). On day 3, fewer but larger UMOD-positive casts were seen throughout the tissue (Fig. 3d). After 8 weeks, the distribution pattern was almost that of controls. (Fig. 3e). In addition, in some tubules a shift in UMOD staining before (Fig. 3f) and after I/R (Fig. 3g) could be observed from the apical membrane to the basolateral side as illustrated by the profile of fluorescence signals of cross-sections of two representative tubules (Fig. 3f–i). The control sample clearly showed two peaks—one on each apical side of the lumen (Fig. 3h). In contrast, the 24 h post-reperfusion sample showed four signal peaks—two on each side of the tubule—representing one apical and one basolateral signal (Fig. 3i).

**Fig. 4:**
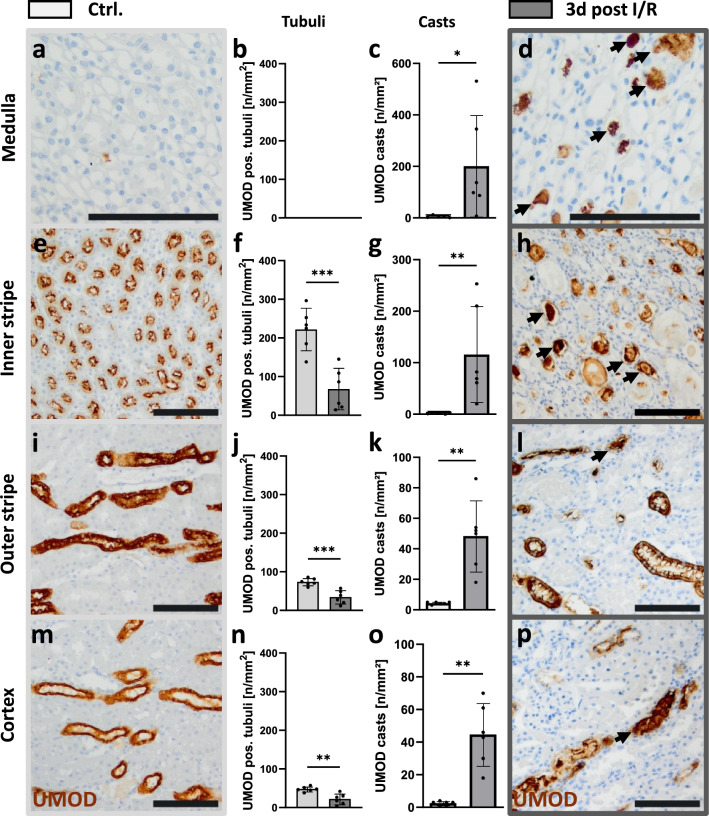
UMOD levels in different renal compartments after I/R injury. UMOD expression in the kidney of sham-operated rats (Ctrl., **a**, **e**, **i**, **m**) compared to I/R-injured kidneys (**d**, **h**, **l**, **p**) as assessed by immunohistochemistry (brown staining). The number of uromodulin positive tubuli (**b**, **f**, **j**, **n**, left) and the number of UMOD-positive intratubular protein casts (**c**, **g**, **k**, **o**, right) were evaluated in the distinct renal regions, separately and examples of casts are marked by arrows (**d**, **h**, **l**, **p**); (* *P* < 0.05; *** *P* < 0.001); Scale bar = 100 µm

**Fig. 5:**
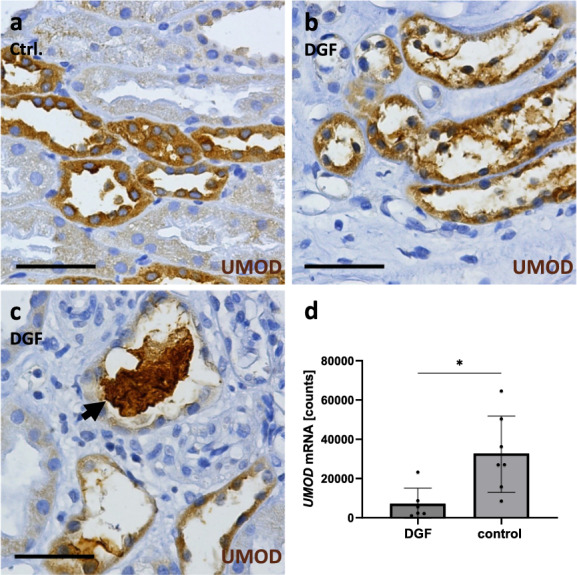
UMOD staining and *Umod* gene expression after kidney transplantation in humans. UMOD was stained brown by immunohistochemistry in 1-year protocol kidney biopsies with no apparent kidney damage (**a**, Ctrl.) and in kidney transplant biopsies with DGF within the first 2 weeks after transplantation showing disrupted UMOD expressing tubules (**b**) and UMOD-positive casts (**c**, arrow). Number of *Umod* mRNA molecules assessed by gene expression analysis in biopsies taken 2 weeks (DGF) and 57 weeks after transplantation (control) (**d**). Scale bar = 50 µm

The compartment specific UMOD levels were further quantified on day 3 after I/R using semi-quantitative scoring of UMOD immunohistochemistry and distinguishing between UMOD-positive cells and casts (Fig. 4). In healthy animals, UMOD was absent in the medulla (Fig. 4a, b). In contrast, UMOD positive material could be detected in the medulla of ischemic kidneys, and was restricted to protein casts (Fig. 4c, d). The highest abundance of UMOD-expressing tubuli was seen in the inner stripe of healthy rats (Fig. 5e, f); this was reduced to one third in I/R-injured animals (Fig. 5f, h). In the outer stripe and cortex, the frequency of UMOD-positive tubules was lower compared to the inner stripe but also significantly reduced compared to healthy controls (Fig. 4i, j, l, m, n, p). In contrast, UMOD-positive casts were also found in the inner stripe (Fig. 4g), the outer stripe (Fig. 4k) and the renal cortex (Fig. 4o) of I/R kidneys but were not found in any kidney compartment of healthy rats (Fig. 4c, g, k, o).

### Severe damage of UMOD-producing tubules with cast formation in human kidney grafts after transplantation

In humans, processes similar to those in the I/R rat model occur shortly after kidney transplantation. Biopsies from kidney grafts with delayed graft function (DGF), taken within the first 2 weeks after transplantation, revealed severe tubular injury, especially in UMOD-positive tubular segments, compared to 1-year protocol biopsies serving as controls (Fig. 5a, b). UMOD-expressing tubular epithelia were either atrophied or completely destroyed, and UMOD-positive casts were frequently observed (Fig. 5b, c). Similarly, *Umod* mRNA expression was at a low level in biopsies with DGF and showed a significant increase one year later in the same grafts (Fig. 5d).

### Serum UMOD levels in CKD depend on nephron number

**Fig. 6:**
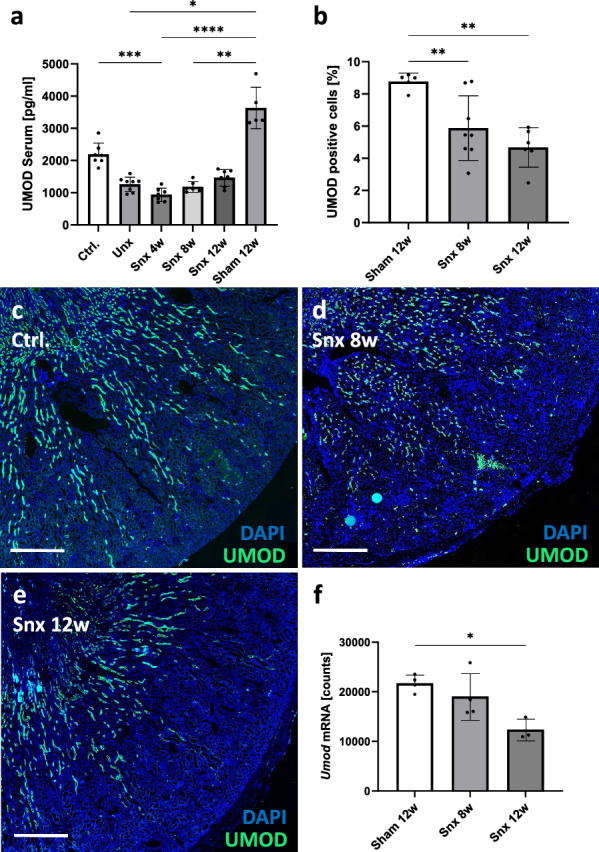
Serum and renal UMOD protein and *Umod* gene expression analysis in Unx and Snx rats. Serum UMOD was measured using ELISA in healthy controls (Ctrl.), uni-nephrectomized (Unx) or 5/6-nephrectomized (Snx) (**a**); Percentage of UMOD positive cells (**b**) in rat kidneys taken at different time points after Snx were analyzed by computer-assisted cell counting using QuPath software; Examples for fluorescence staining at different time points (**c–e**); Number of renal *Umod* mRNA molecules assessed by gene expression analysis (**f**). Staining: blue = nuclei; green = UMOD; (* *P* < 0.05; ** *P* < 0.001); Scale bar = 1 mm

To test the hypothesis that serum UMOD concentrations in CKD depend on the number of nephrons, we investigated healthy rats compared to Unx and Snx rats. Circulating UMOD correlated positively with the number of nephrons within the first 4 weeks after Snx, showing the highest level in healthy rats, which was significantly reduced by 42.4 ± 9.5% after Unx and further declined to 57.2 ± 8.8% four weeks after Snx (Fig. 6a). However, when sera from rats that underwent Snx were examined after 8 and 12 weeks, UMOD concentrations gradually restored and reached levels of Unx animal in week 12 (Fig. 6a).

The slight increase in serum UMOD during the course of Snx is surprising, as both the percentage of UMOD-positive cells (Fig. 6b) and the *Umod* expression (Fig. 6f) decreased as renal disease progressed during the 12 weeks after Snx. *Umod* gene expression correlated with the number of UMOD-positive cells, but not with circulating UMOD (Table 2). The decrease of UMOD-positive cells starting from 8.7 ± 0.5% in controls to 5.9 ± 1.9% at 8 weeks and 4.7 ± 1.1% at 12 weeks after induction of Snx (Fig. 6b) suggests a progressive loss of nephrons, where the distribution of UMOD-positive tubules, membrane debris, and casts appeared to change with nephron loss. In sham-operated rats, tissue related UMOD occurred predominantly in the inner and outer stripes and distributed in a radial way (Fig. 6c). At 8 weeks after Snx, UMOD-positive cells were spread throughout the medulla, inner and outer stripes and cortex (Fig. 6d). In kidneys examined 12 weeks after Snx, hardly any UMOD-positive material remained in the cortex, this was mainly confined to the inner stripe with some small radial extensions in the outer stripe (Fig. 6e).

**Table 2:**
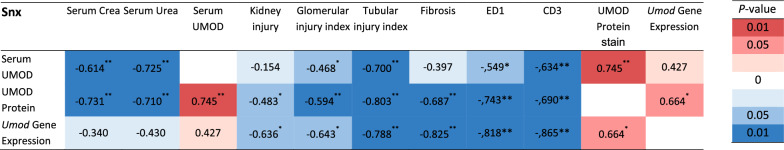
Overview of the correlation between UMOD/*Umod* expression, kidney injury, fibrosis and inflammation in progressing Snx

The correlation coefficients of all correlation analysis between results for serum UMOD, UMOD protein (stain of UMOD-positive cells detected by immunofluorescence) and *Umod* gene expression and evaluations of kidney injury, fibrosis and inflammation were summarized and displayed in a heat map; n = 10–35 (* *P* = 0.05; ** *P* = 0.001). Positive correlations are colored red, negative correlations blue, and highly significant correlations are colored deeper

### Decrease of UMOD protein and gene expression was associated with decreased kidney function and increased tubular injury and inflammation

**Fig. 7:**
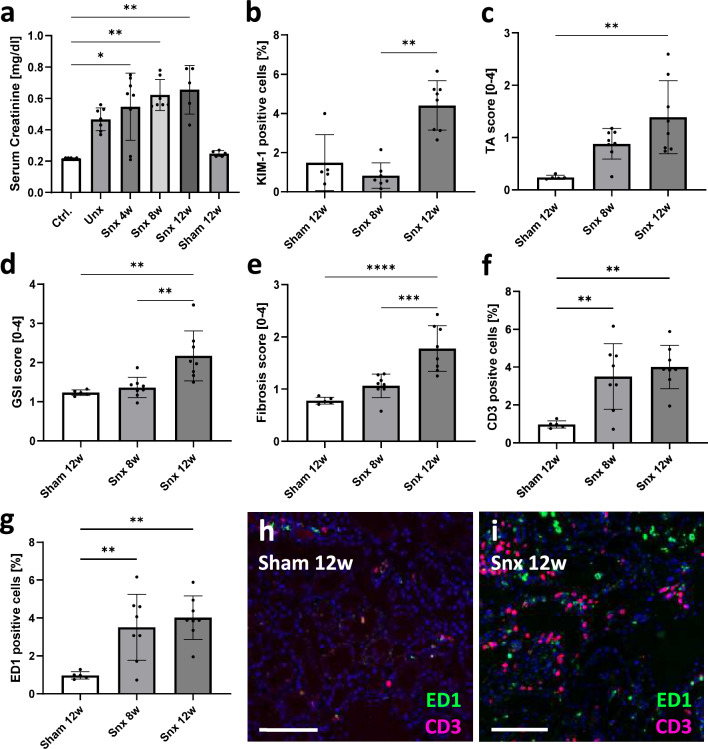
Analysis of rat kidney function, injury, fibrosis and inflammation after 5/6-nephrectomy (Snx). Kidney function was assessed by serum creatinine [mg/dl] (**a**); Kidney injury was measured as percentage of KIM-1 positive cells in IF staining (**b**); Tubular atrophy (TA, **c**), glomerulosclerosis (GSI, **d**) and fibrosis (**e**) was assessed by scoring of histological changes; percentage of CD3 positive T-cells (**f**) and ED1 positive macrophages (**g**) in rat kidneys taken at different time points after Snx were analyzed by computer-assisted cell counting using QuPath software; Examples of fluorescence staining at 12 weeks after sham operation (**h**) and Snx (**i**) are shown. Scale bar = 100 µm; (* *P* < 0.05; ** *P* < 0.01; *** *P* < 0.001; **** *P* < 0.0001)

The decrease in renal UMOD and *Umod* gene expression 8 and 12 weeks after Snx paralleled a decline in kidney function, indicated through increased serum creatinine (Fig. 7a), kidney injury (KIM-1-positivity) (Fig. 7b), tubular atrophy (Fig. 7c), glomerulosclerosis (Fig. 7d) and interstitial fibrosis (Fig. 7e). In addition, the number of CD3-positive T-lymphocytes and macrophages in the kidney tissue was 3–4 times higher in Snx rats compared to sham-operated controls (Fig. 7f–i). Correlation analyses confirmed that lower serum UMOD was associated with an elevated tubular injury index (r = -0.700), increased serum creatinine (r = -0.614) and urea (r = -0.725), but also with inflammation, especially T-cellular (Table 2). Circulating UMOD was positively associated with quantification of UMOD-positive cell number in the kidney (r = 0.745), but not with gene expression (Table 2). *Umod* gene expression correlated negatively with tubular injury (r = -0.788), interstitial fibrosis (r = − 0.825) and inflammatory infiltrates like CD3-positive T cells (r = − 0.865) and ED1-positive macrophages (r = − 0.818) (Table 2).

## Discussion

### Increased serum UMOD as an early biomarker for ischemic acute kidney injury

In our animal study of transient ischemia (I/R), we demonstrated that serum UMOD increased significantly as early as 6 h after induction of tissue damage. This early increase preceded the rise in serum creatinine, suggesting a superior diagnostic sensitivity of serum UMOD. This has also been demonstrated in patients with very early forms of CKD [12, 16]. The use of serum UMOD as an early biomarker of acute kidney injury (AKI) allows for earlier therapeutic intervention, potentially reducing damage. In contrast, previous work in mice reported a parallel rise of UMOD and creatinine, which did not start until 24 h after induction [21]. It should be noted that induction of I/R in our rat model resulted in more severe tubular damage than previously published [21] with more pronounced depletion of UMOD-positive tubular epithelia and increase in circulating UMOD. This suggests that the sensitivity of UMOD as a marker may vary across species and models, with our results indicating an earlier increase in the context of more severe tissue damage. The severity of acute tubular injury might influence the time course as well as the amount of serum UMOD, in line with the coincidence of histological tubular damage and increased circulating UMOD. In healthy kidneys, most UMOD is released apically into the tubular lumen while a smaller amount of intracellular UMOD is transported basolateral into the interstitium and blood. Following acute I/R injury epithelia of the TAL are particularly susceptible and can completely disintegrate [28], resulting in sudden release of large amounts of UMOD-positive material from the disrupted and necrotic epithelia, as shown in Figs. 4 and 5. This mechanism observed in our animal models may also be important in humans, particularly in cases of acute kidney injury. In addition, following I/R injury UMOD increasingly translocates to the basolateral epithelial domain and is actively transported into the circulation via the interstitium [11, 21]. This could be confirmed by an intense apical staining in the control group and a stronger basolateral and interstitial reactivity in the 24 h I/R group. Recent studies show that the non-aggregating full-length UMOD form is also present in urine, albeit to a much lesser extent [29]. Backflow of urinary UMOD seems unlikely to enter the blood after AKI because we could not detect cleaved UMOD in the serum 24 h after I/R. In a study investigating post-transplant serum UMOD levels in humans, serum UMOD dramatically rose in patients immediately after transplantation from non or nearly unmeasurable levels [12]. However, as time progressed, serum UMOD levels clearly discriminated between patients who developed immediate graft function (IGF) and those who developed delayed graft function (DGF). Namely UMOD levels remained significantly lower in patients with DGF not reaching a defined cut-off level within the first 10 days as observed in patients with IGF, thus indicating that serum UMOD is a valuable tissue biomarker capable of closely monitoring kidney function [12]. Our present data confirmed downregulation of UMOD on mRNA level and indicate disturbance of UMOD expressing cells in DGF biopsies. However, a very early increase in serum UMOD observed in our acute and severe I/R-model, which theroretically might also be caused I/R injury injury during transplantation, was not observed in this clinical study [12], possibly due to irrigation of the organ with preservative solution to maintain the graft and preconditioning prior to transplantation, which might wash out UMOD released from injured tubular cells before it can be spilled into the circulation of the transplanted recipient.

### Renal UMOD distribution and *Umod* expression reflect renal injury and subsequent regeneration

Although the number of *Umod*-expressing cells characteristically decreased in the first days after I/R, the amount of intratubular UMOD casts was dramatically elevated in the I/R group after 3 d. It is reasonable to assume that cast formation does not exclusively depend on the amount of (soluble) urinary UMOD. This highlights the importance of UMOD as a marker for the severity of TAL cell injury and the associated release of UMOD-positive material [20]. This could be clinically relevant for quantifying the extent of tissue damage and monitoring the healing process in humans. Casts are formed in the tubular lumen by high molecular weight filaments of UMOD [30]. Accordingly, damage to TAL cells was associated with a rapid decline in UMOD-expressing cells, and persistently low numbers of these cells may reflect missing or incomplete repair mechanisms and misadapted cell recovery. Gene expression of *Umod* also strongly dropped 24 h after I/R at the peak of the injury and remained suppressed for several days. Others have conducted I/R experiments with similar long-lasting episodes of ischemia and severity of injury as in our model, showing nearly identical results with a downregulation of *Umod* for several days [31, 32]. Studies with a shorter ischemia time showed only a short downregulation of *Umod* 24 h after I/R, with a faster recovery of *Umod* expression [21]. Similar findings regarding renal U*mod* expression have been reported in other AKI models in which the injury was not induced by ischemia, but e.g. by sodium oxalate treatment [33]. However, these studies did not investigate circulating UMOD levels. The changes in Umod expression and distribution observed in our study may also serve as indicators of damage and healing processes in humans following acute kidney injury.

### Potential anti-inflammatory role of UMOD in renal disease

Beside its nephroprotective characteristics, UMOD can activate inflammatory cells such as neutrophils, macrophages and dendritic cells [34–37], suggesting a role as a danger-associated molecule [38]. However, these results refer to in vitro studies with urinary UMOD, and remains unclear whether basolaterally released UMOD also modulates inflammation [37]. In vivo studies with UMOD deficient mice showed increased inflammation and worse kidney function after I/R [20, 21], which in turn indicates an immuno-protective role of UMOD. This suggests that a prolonged lack of UMOD during AKI, as it occurs after more than 30 min ischemia, leads to more severe and persistent inflammation [39]. An immunoregulatory function of UMOD is also suggested by our observations in the Snx model of chronic kidney disease, where low UMOD serum levels and *Umod* gene expression in the kidney correlated with increased inflammation. This dual role of UMOD as a nephroprotective factor and potential trigger of inflammation is highly relevant for understanding the pathophysiology of human kidney diseases.

### Serum UMOD levels depend on nephron number and can be modulated by increased basolateral transport

We demonstrated that circulating UMOD decreased with the number of nephrons and—different to AKI—correlated negatively with kidney function and injury. This correlation may be significant in humans for assessing the degree of nephron loss and the progression of chronic kidney disease (CKD). Additionally, the amount of UMOD protein was reduced with lower nephron number and decreased progressively between weeks 8 to 12 after Snx. These results suggest that during progressive CKD nephron loss leads to a decline of the GFR and absolute UMOD production [15, 16] and might be self-perpetuating. It is generally accepted that the functional capacity of the kidney is proportional to the GFR, which in turn depends on the number of nephrons, known to decline with age in healthy adults [40]. Loss of nephrons and inflammatory response are pivotal processes in the development of CKD and both lead to fibrosis [41]. Interestingly, serum UMOD is a more sensitive marker for early-stage CKD than serum creatinine, urea or cystatin C [16] and is much less prone to degradation than urinary UMOD [42]. Several studies suggested that the reduced number of UMOD producing tubular cells, caused by nephron loss, results in low serum levels of UMOD [17, 43]. After removal of one kidney (Unx), serum UMOD levels in our rat model were almost halved (to 58%). In contrast, a human study in living donors showed only a 33% reduction in serum UMOD levels [19]. However, there the circulating UMOD of living donors was not measured immediately after nephrectomy, but 9–20 months later. Therefore, it cannot be ruled out that serum UMOD levels were halved immediately after the nephrectomy, but already underwent compensation. In our animal experiments we made similar observations: After additional nephron mass reduction by Snx, serum UMOD decreased further within the first 4 weeks, but slightly increased again 12 weeks after Snx, as CKD progressed. While kidneys cannot generate de novo nephrons, existing nephrons can expand dramatically. In the I/R model the UMOD-producing cells regenerated after some days and serum UMOD returned to stable levels after the transient increase during the acute injury. In contrast, in the Snx model the remaining nephrons were not initially damaged but were increasingly stressed by hyperfiltration and inflammation. Both the number of UMOD-expressing cells and *Umod* gene expression decreased with the progression of renal injury while UMOD serum levels did not decline, again suggesting a shift in UMOD transport from mainly apical to basolateral cell site. The stimuli regulating the expression and changes in UMOD transport in renal disease are still unknown. Nevertheless, this observation may help to better understand the relationship between nephron loss and UMOD expression in humans, which is important for the development of biomarkers for CKD.

Further, upregulation of UMOD has also been described in early diabetes mellitus with hyperfiltration lacking morphological visible glomerular injury [44, 45], and in sepsis [46].

Part of our study is limited by the use of rat models. Rats have a smaller number of nephrons compared to humans, and the number of nephrons also varies widely among healthy humans [47]. This limitation should be considered when extrapolating these results to humans, as differences in nephron number and variability could affect the outcomes. Furthermore, it remains unclear if AKI induced by toxic insults also leads to an early increase in UMOD serum levels, as reduced tissue staining of the marker has been observed in sodium oxalate induced AKI [33].

Taken together, we have shown that a sudden increase of circulating UMOD due to I/R stress may be a useful biomarker for early diagnosis of AKI, allowing for earlier therapeutic intervention, while decreasing UMOD levels reflect chronic nephron loss. These findings emphasize the potential of UMOD as a useful diagnostic biomarker for different stages and types of kidney injury in humans.

## Data Availability

The datasets generated and/or analysed during the current study are available in the figshare repository, https://doi.org/10.6084/m9.figshare.26115160.

## Abbreviations

AKI: Acute kidney injury
ATI: Acute tubular injury
CKD: Chronic kidney disease
DA: Dark Agouti
DGF: Delayed graft function
I/R: Ischemia/reperfusion
PAS: Periodic acid-Schiff
Snx: 5/6-Nephrectomy
TAL: Thick ascending limb
UMOD: Uromodulin
Unx: Uninephrectomy
